# Pure-Tone Hearing Thresholds and Otoacoustic Emissions in Students of Music Academies

**DOI:** 10.3390/ijerph18031313

**Published:** 2021-02-01

**Authors:** Małgorzata Pawlaczyk-Łuszczyńska, Małgorzata Zamojska-Daniszewska, Adam Dudarewicz, Kamil Zaborowski

**Affiliations:** Department of Physical Hazards, Nofer Institute of Occupational Medicine, 91-348 Lodz, Poland; zamojska@interia.pl (M.Z.-D.); adam.dudarewicz@imp.lodz.pl (A.D.); kamilzaborowski@op.pl (K.Z.)

**Keywords:** noise-induced hearing loss, music students, exposure to excessive sounds, pure-tone audiometry, hearing threshold levels, high-frequency notches, transient evoked otoacoustic emission (TEOAE), distortion product otoacoustic emission (DPOAE)

## Abstract

The objective of this study was to assess the hearing of music students in relation to their exposure to excessive sounds. A standard pure-tone audiometry, transient-evoked otoacoustic emissions (TEOAEs) and distortion-product otoacoustic emissions (DPOAEs) were determined in 163 students of music academies, aged 22.8 ± 2.6 years. A questionnaire survey and sound pressure level measurements during solo and group playing were also conducted. The control group comprised 67 subjects, mainly non-music students, aged 22.8 ± 3.3 years. Study subjects were exposed to sounds at the A-weighted weekly noise exposure level (L_EX,w_) from 75 to 106 dB. There were no significant differences in the hearing thresholds between groups in the frequency range of 4000–8000 Hz. However, music students compared to control group exhibited lower values of DPOAE amplitude (at 6000 and 7984 Hz) and signal-to-noise ratio (SNR) (at 984, 6000, and 7984 Hz) as well as SNR of TEOAE (in 1000 Hz band). A significant impact of noise exposure level, type of instrument, and gender on some parameters of measured otoacoustic emissions was observed. In particular, music students having L_EX,w_ ≥ 84.9 dB, compared to those with L_EX,w_ < 84.9 dB, achieved significantly lower DPOAE amplitude at 3984 Hz. Meanwhile, both TEOAE and DPOAE results indicated worse hearing in students playing percussion instruments vs. wind instruments, and wind instrument players vs. students playing stringed instruments.

## 1. Introduction

Excessive exposures to loud music may cause various hearing symptoms (e.g., tinnitus or temporary threshold shift of hearing) and consequently lead to noise-induced hearing loss (NIHL). Professional musicians due to chronic exposure to loud sound (music) and long impact during the day are more predisposed to the development of hearing loss as compared to those who listen to music only occasionally [1,2,3,4,5,6,7,8,9,10,11,12,13].

Recently, Di Stadio et al. [13] conducted a systematic review of literature aimed at evaluation of the risk of hearing loss and prevalence of hearing symptoms among professional musicians. Analysis of 41 articles describing the results of research in the group of 4618 professional musicians, aged from 18 to 69 years, representing various types of music, indicates that hearing loss occurred in 38.6% of the tested subjects. Hearing impairment was found nearly twice as frequently in rock or pop musicians than in classical musicians (63.5% vs. 32.8%, *p* < 0.05) and mainly affected the range from 3000 to 6000 Hz. The most often observed hearing symptoms were tinnitus (26.3%), hyperacusis (21.7%) and diplacusis (6.3%). Classical musicians and rock or pop musicians equally often complained about tinnitus. On the other hand, hyperacousis was more often found among pop or rock musicians, whereas diplacusis in classical musicians [13].

Although the studies on potential harmful effects of music on hearing have been conducted for over half a century, there are still different opinions and speculations as to the risk of hearing impairment caused purely by exposure to music [10]. The results of some of the studies indicate that musicians’ hearing thresholds are higher (worse) than would result from age (and gender), which suggests that the hearing defects were caused by music and they associate the risk of hearing impairment with its level and duration [1,3,4,7,8,9]. Instead, other studies imply that music impairs hearing to a lower extent than its level indicates [2,5,6]. In particular, some researchers [11,12] suggest that orchestral noise deteriorates hearing less than expected from sound exposure according to the ISO 1999:2013 model [14].

Furthermore, most of the earlier studies concerning the risk of NIHL due to exposure to loud music were mainly focused on professional musicians, employees of musical clubs and people who often listen to loud music [10]. Considerably less attention was paid to students of music schools who in view of their future career of professional musicians constitute a group of increased risk. However, some studies provide evidence that students of academies of music, similarly to professional musicians, are often exposed to sound at high levels (above 85 dBA) creating a risk of hearing impairment [15,16,17,18]. This is demonstrated both by the results of sound pressure level measurements during individual and collective classes and the attempts to evaluate the daily exposure based on full-day measurements using noise dosimeters, involving a typical schedule of students’ classes [19,20,21].

The golden standard in diagnosis of NIHL is a standard pure-tone audiometry (PTA). However, this test enables detection of the hearing loss no sooner than when the cochlea damage is irreversible [22]. An alternative for pure-tone audiometry can be the measurement of otoacoustic emissions (OAEs), especially the transient-evoked otoacoustic emission (TEOAE) and distortion-product otoacoustic emission (DPOAE), since they can give information about weakened function of cochlea before the problems are seen in audiograms.

Otoacoustic emissions are week acoustic signals generated in the inner ear and registered in the outer ear, whose measurement is used as an objective hearing test. They occur in response to an acoustic stimulus or spontaneously. OAE measurement has been proposed as an objective and sensitive method of detecting preclinical damage of the cochlea due to noise exposure and monitoring early signs of NIHL [22]. Whether TEOAEs or DPOAEs can be applied as diagnostic tools for musicians or not has not been adequately established. Nevertheless, the measurements of both types of OAEs were considered to be valid and reliable to be used in the hearing conservation programmers for monitoring individuals [8,23,24]. Furthermore, OAE investigations were previously used by some researchers to study the change in cochlea due to music exposure in various groups of musician, in particular among classical orchestral musicians and rock musicians.

For example, Emmerich et al. [8] measured audiometric HTLs and DPOAEs in a group of 109 professional orchestral musicians (aged 30–69 years) and found that their hearing threshold levels become higher (worse) and OAE amplitudes decline with the duration of employment.

Since noise-induced hearing loss develops slowly, it is unlikely that young music students’ hearing would be deteriorated. Thus, to identify possible early signs of NIHL due to regular playing instruments, extended high-frequency audiometry and/or otoacoustic emissions may be applied and the prevalence of high-frequency notched audiograms among music students can be analyzed.

For example, Lüders et al. [25] have analyzed hearing threshold levels from 0.25 to 16 kHz in a group of 42 music students in comparison to a non-musician group in order to determine if high-frequency audiometry is a useful tool in the early detection of hearing impairment. When tested using conventional audiometry, a majority of subjects (92.9%) had hearing thresholds within normal limits. Nevertheless, both standard PTA and extended high-frequency audiometry (EHFA) revealed significantly higher (worse) hearing threshold levels in the music students as compared to the control group. Furthermore, the worst results occurred at 0.25, 6, 9, 10, and 11.2 kHz, thus confirming that EHFA may be useful in the early detection of hearing loss in musicians.

On the other hand, Phillips et al. [26] having analyzed audiograms of 329 classical music students (aged 18–25 years) in view of the prevalence of early signs of NIHL found in 45% of them high frequency notches (≥15 dB depth), mainly occurring at 6 kHz. Although notched audiograms occurred more frequently in students who individually practiced playing instruments for at least 2 h daily, no correlation was found between the incidence of notches and the type of instrument or exposure to sounds. [26]. In another study, carried out by Barlow [27], 44% of 50 young people studying popular music showed evidence of audiometric notches at 4000 or 6000 Hz, and 16% exhibited a moderate hearing loss. No correlation was found between the age and presence of notches. Their occurrence was equally probable in younger as in adult students.

More recently, Henning and Bobholz [28] compared the results of the DPOAE and PTA measurements in the group of 28 college music majors and 35 non-music majors enrolled at a university with normal hearing (aged 18–25 years). They showed that, despite the lack of significant differences between the groups in the pure-tone audiometry, the students of music faculties achieved worse results of DPOAE testing. In particular, significantly greater prevalence of absent DPOAEs as well lower (but not significantly) amplitudes of recorded signals were noted in music majors as compared to non-music majors.

Earlier, Gopal et al. [20] demonstrated that the college music students participating in jazz band-based instructional activity were exposed to much higher A-weighted equivalent continuous sound pressure levels, as compared to non-music faculty students, during 50-min classes. Consequently, they exhibited a significant post-exposure temporary threshold shift (TTS) bilaterally at 4000 Hz as well as a significant temporary decrease in the amplitude of TEOAEs after termination of those classes, while, in the group of non-music faculty students, such changes were not found.

Thus, the overall purpose of the present study was to evaluate the hearing status of ungraduated classical music students in relation to their regular exposure to sound (music) due to playing instruments. In particular, this has been attempted to:

- compare music students as regards hearing with their peers who were not occupationally exposed to noise and did not play music instruments, and answer whether or not playing instruments is associated with a higher risk of NIHL,
- examine if intensity of music exposure or the type of instrument being played had an impact on hearing status evaluated using conventional pure-tone audiometry as well as transient-evoked otoacoustic emissions and distortion-product otoacoustic emissions.

## 2. Materials and Methods

Hearing tests and questionnaire surveys were conducted among undergraduate classical music students to assess their hearing status and to gather data on their musical experience, instruments in use, time of weekly playing and to identify other—apart from regular excessive exposure to sound—risk factors for NIHL. In parallel, sound pressure level measurements were made in music students while they were playing instruments.

The study group comprised 168 females and males, aged 19–32 years, who study at two Polish academies of music. The control group consisted of 67 women and men, aged 18–31 years, mainly non-music students who were not occupationally exposed to noise and did not play any musical instruments.

Participation in the research was voluntary. Subjects were recruited by advertisement. The course of the experiment and applied research methods were approved by the Ethic Committee of the Nofer Institute of Occupational Medicine of Lodz, Poland (decision no. 8/2013). The participants obtained some remuneration and certified in writing their consent to participate in the research.

### 2.1. Hearing Tests

All subjects underwent standard PTA and OAE measurements, specifically transient- evoked otoacoustic emissions and distortion-product otoacoustic emissions. The auditory rest before hearing tests lasted 14 h. Prior to the audiological evaluations, otoscopy was performed. Only subjects who met the inclusion criteria, i.e., a normal otoscopy picture, lack of a history of chronic ear diseases, head injury and ototoxic drugs were included in the study.

Standard pure-tone audiometry was conducted with the VIDEOMED Smart Solution (Szczawno-Zdrój, Poland) clinical audiometer, model AUDIO 4002 with the Holmberg GMBH & Co. KG Electroacoustics (Berlin, Germany) headphones type HOLMCO PD-81. Hearing threshold levels (HTLs) for air conduction were determined using the ascending–descending technique in the 5-dB steps at frequencies from 0.250 to 8 kHz.

Mean values of HTLs were compared in subgroups of the study subjects. The prevalence of normal audiograms, high- and speech-frequency hearing losses, as well as high-frequency notched audiograms were also analyzed in the study subjects (ears). Normal hearing was defined as having HTLs between 0.25 and 8 kHz ≤ 20 dB HL. The speech- and high-frequency hearing loss was defined as the pure-tone mean of >20 dB HL at 0.5, 1, 2 and 4 kHz, and 3, 4 and 6 kHz, respectively. In turn, the notch was defined as a sharp drop in the hearing acuity at 4 or 6 kHz of at least 15 dB in relation to both the best preceding threshold occurring at frequencies from 1 to 3 (4) kHz and the threshold at 8 kHz.

A Scout Otoacoustic Emission System ver. 3.45.00 (Bio-logic System Corp., One Bio-logic Plaza, Mundelein, IL, USA) was applied for recording and analyzing of otoacoustic emissions. For TEOAE measurements, a standard click stimuli at sound pressure levels (SPL) of about 80 dB were generated. Each response was windowed from 3.5 to 16.6 ms post stimulus and band-pass filtered from 0 to 6000 Hz. The total number of stimuli was 260. The artefact rejection level was set at 20 mPa. The amplitude and reproducibility of response as well as the noise floor amplitude during the recordings and corresponding signal-to-noise ratio (SNR) were determined for the overall frequency range and for ½-octave bands with central frequencies of 1, 1.5, 2, 3 and 4 kHz. The signal-to-noise ratio >6 dB and reproducibility >60% were adopted as the criteria of the TEOAE presence.

For DPOAE testing, a stimulus in the form of a two-tone was used with the fixed ratio of frequencies f_1_ and f_2_ (f_1_/f_2_ = 1.22) and the intensity levels L_1_ and L_2_ of 65 and 55 dB SPL, respectively. The amplitudes of registered signals were determined at the 2f_1_–f_2_ frequencies as a function of f_2_ frequencies (ranged from 750 to 7968 Hz in 1/2-octave intervals) together with the noise floor and corresponding SNR. The DPOAE signals were considered as present if the signal-to-noise ratio at a particular frequency pair was greater than 6 dB.

The presence and absence of TEOAEs and DPOAEs were analyzed in both study groups and their subgroups. In particular, the proportions of subjects with absent OAEs for at least one frequency band (or frequency) in at least one ear were evaluated. The mean values of the TEOAE and DPOAE amplitudes and signal-to-noise ratios as well as reproducibility of TEOAEs were also compared in subgroups of examined subjects. When analyzing the TEOAE and DPOAE amplitudes, two variants of calculations were performed, i.e., without and with exclusion data not meeting the criterion of SNR > 6 dB.

Hearing examinations were carried out by the same investigator in sound-proof cabin or in quiet rooms located in academies’ buildings where the A-weighted equivalent-continuous sound pressure level of background noise did not exceed 35 dB.

### 2.2. Questionnaire Surveys

All music students filled in a special questionnaire developed to gather, first of all, data on their musical experience, instruments in use, time of weekly practice as well as to identify other, apart from frequent playing instruments, risk factors for noise-induced hearing loss.

This questionnaire included the questions about: (a) demographic data, (b) exposure to sounds while playing musical instruments (period of education/playing instruments, type of instruments, time devoted to individual or collective playing during obligatory and additional classes, rehearsals and performances, etc.), (c) self-evaluation of physical health and medical history (past middle-ear diseases, and surgery, etc.), (d) physical features (body weight, height, skin pigmentation), (e) lifestyle (smoking, noisy hobbies, listening to personal media player, attending disco/bars, rock concerts etc.), (f) use of individual hearing protectors, and (g) self-assessment of hearing status. Subjects from the control group were also interviewed using a similar questionnaire, but without questions on music exposure.

In addition, all subjects completed a modified Amsterdam Inventory for Auditory Disability and Handicap (AIADH). This questionnaire is divided into five parts (subscales) assessing: (a) ability of discrimination (differentiation) of sounds (subscale I), (b) auditory localization (subscale II), (c) understanding speech in noise (subscale III), (d) intelligibility in quiet (subscale IV), and (e) detection of sounds (subscale V) [29]. However, the outcomes of this questionnaire were presented in the other paper [30].

### 2.3. Evaluation of Exposure to Sounds

To evaluate music students’ exposure to excessive sounds (so called “music noise”) sound pressure levels (SPLs) were measured during individual and ensemble playing at the academy, during classes with the teacher or rehearsals and concerts. These surveys included various instruments and diverse repertoire.

The measurements were performed according to Polish standards PN-N-01307:1994 and PN-EN ISO 9612:2011 [31,32] using personal sound exposure meters (i.e., the Brüel & Kjær, personal logging dosimeters type 4436 and 4443) worn by the students playing instruments. The microphone of each dosimeter was usually mounted on the top of the shoulder of what a young musician judged to be his or her most exposed ear (approx. 0.04 m above the shoulder). Care was also taken not to disturb playing instrument. The distance between microphone and the ear ranged from 0.10 to 0.40 m. For example, violinists always had the microphone mounted on their right shoulder.

Each single measurement period usually corresponded to the duration of rehearsal, lesson, or concert. Altogether, 294 noise samples (covering in total approx. 231 h) were collected.

For each music student, the A-weighted weekly noise exposure level (L_EX_, _w_) was calculated from the values of the A-weighted equivalent-continuous sound pressure levels produced by the respective instrument (e.g., violin or trumpet) and time of weekly (solo and group) practice obtained from the questionnaire, using the following Equation (1):

(1) $$L_{\mathrm{EX},\;w}=\;10\;\cdot \log\;\left[\frac{1}{T_{o}}{(T}_{1}\cdot 10^{0.1\cdot L_{\mathrm{Aeq},T1}}+T_{2}\cdot 10^{0.1\cdot L_{\mathrm{Aeq},T2}})\right]$$

where L_Aeq,T1_ is (the energy average of collected samples of) the A-weighted equivalent-continuous SPL produced by respective instrument during collective playing, in dB; L_Aeq,T2_ is (the energy average of collected samples of) the A-weighted equivalent-continuous SPL produced by respective instrument during individual playing, in dB; T_1_, T_2_ are the declared times of group and individual practice per week, in hours; T_o_ is the reference duration, T_o_ = 40 h.

In case of students playing various instruments, the L_EX, w_ levels were calculated taking into account sound pressure levels assigned to their main instruments in use.

### 2.4. Statistical Analysis

Differences between music students and the control group in results of hearing tests (e.g., mean values of the TEOAE amplitudes) were evaluated using the *t*-test for independent samples or—if the preconditions of its use were not met—the Mann–Whitney-U test was applied. Similar tests were used for comparison, in both groups, of the mean values of other variables (e.g., average age). In turn, differences between right and left ears in the hearing test results were assessed using the *t*-test for dependent samples or Wilcoxon signed-rank test, when applicable.

Frequency of specific answers given to the questionnaire in various subgroups of study subjects as well as prevalence of some outcomes of hearing tests (e.g., incidence of absent DPOAEs or notched audiograms) were presented as proportions with 95% confidence intervals (95% CI), while the differences between them were compared in pairs using the chi-square test.

The main effects analysis of variances (ANOVA) was used to evaluate the first-order (non-interactive) effects of multiple factors such as: gender, age and noise exposure or type of instrument on the hearing test results (e.g., the amplitude of TEOAEs). For this purpose, the group of music students was divided into subgroups according to gender (women and men), age (younger and elder individuals), and exposure to music (lower- and higher exposed subjects) or the type of instrument (players of percussion, wind and stringed instruments). Median values of age and weekly noise exposure level were used as the basis for subject classification. Differences between the aforesaid subgroups of music students were assessed using the post-hoc Tukey HSD test (including the Tukey HSD test for unequal N) or Taman test (if the assumption of variance homogeneity was not met). The main effects ANOVA were also applied to evaluate the impact of age, gender and type of instrument on students’ exposure to music (i.e., the L_EX,w_ levels).

The STATISTICA (version 9.1. StatSoft Inc., Tulsa, OK, USA) was used for statistical analysis. All tests were conducted with the assumed significance level *p* < 0.05.

## 3. Results

### 3.1. Study Groups Characteristics

The entire study group comprised 168 classical music students, but a few subjects with incomplete results of hearing tests and one student having single sided deafness were excluded from further analysis. In the final study group of 163 students, 97 (59.5%) of the persons played stringed instruments, 55 (33.7%) played wind instruments, and 11 (6.5%) played percussion instruments. That group consisted of 80 (49.1%) women and 83 (50.9%) men, aged 22.8 ± 2.6 years (mean value (M) ± standard deviation (SD)). These persons played musical instruments for the period from 2 to 20 years (M ± SD: 11.9 ± 3.7 years).

The control group consisted of 67 persons who were not occupationally exposed to noise and did not deal occupationally with music, including 30 (44.8%) women and 37 (56.2%) men, aged 22.8 ± 3.3 years (M ± SD). A substantial majority (85.4%) of that group were non-music students. About one-quarter (25.4%) of them were occasionally exposed to noise at their workplace or during practical traineeship.

Generally, there were no significant differences in age and gender between music students and controls (*p* < 0.05). Similar relations were observed when analyzing the self-assessment of physical health, medical history, physical features and some aspects of lifestyle, such as smoking habits and noisy hobbies (shooting, paintball, motor sports, etc.) (*p* > 0.05) However, a higher percentage of young musicians, as compared to the control group, used every day the personal multimedia players (57.1% (95% CI: 49.4–64.4%) vs. 35.8% (95% CI: 25.4–47.8%), *p* < 0.05) and often visited pubs (12.3% (95% CI: 8.0–18.3%) vs. 1.5% (95% CI: 0.0–8.9%), *p* < 0.05). On the other hand, a higher percentage of people from the control group, as compared to musicians, declared a frequent use of noisy tools (41.8% (95% CI: 30.8–53.7%) vs. 28.0% (95% CI: 20.7–34.0%), *p* < 0.05), while a reverse relation occurred in the past (11.9% (95% CI: 6.00–22.2%) vs. 27.0% (95% CI: 20.8–34.3%), *p* < 0.05).

Regarding the prevalence of other NIHL risk factors such as smoking, elevated blood pressure, diabetes, white-finger syndrome, light skin pigmentation, and ototoxic antibiotic treatments, there were no significant differences between music students and control. Furthermore, most of these factors were noted merely in several percent of the examined people. The only exceptions were: cigarettes smoking and fair complexion. The light skin pigmentation was reported by 28.8% (95% CI: 22.4–36.2%) of young musicians and 23.9% (95% CI: 15.2–35.5%) of non-musicians. On the other hand, 35.6% (95%CI: 28.7–43.2%) of music students and 32.8% (95% CI: 22.8–44.8%) of controls declared cigarettes smoking.

### 3.2. Evaluation of Exposure to Music

**Figure 1:**
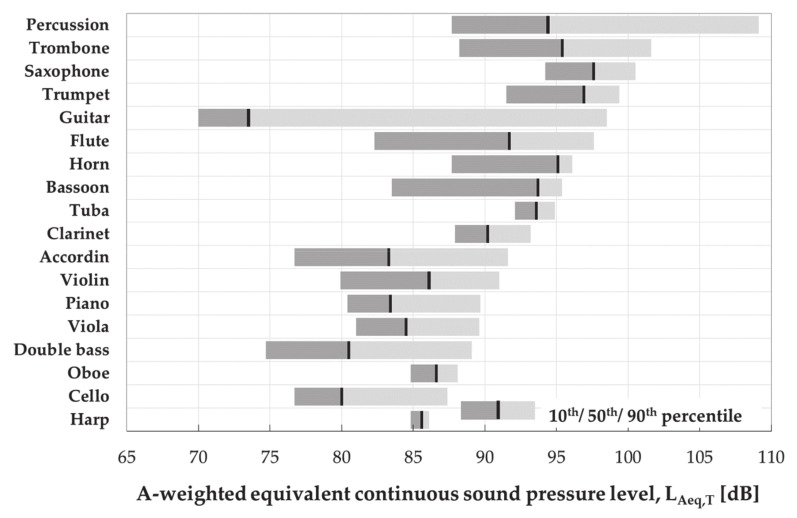
A-weighted equivalent continuous sound pressure levels measured in students playing particular types of instruments during both individual and team practice including various activities, classes with teachers, concerts, etc.

The results of the sound pressure level measurements during individual and group instrument playing were summarized in Table 1 and Table 2 as well as presented in Figure 1. According to the collected data, students were exposed to music noise at (a) the A-weighted equivalent continuous SPL (L_Aeq,T_) of 80–98 dB (10th–90th percentile), (b) A-weighted maximum SPL (L_Amax_) of 94–113 dB, (c) C-weighted peak SPL (L_Cpeak_) of 115–137 dB.

There was a considerable diversity in sound exposure among music students playing various instruments, partly due to variability in the repertoire, kind of lessons and place of testing, etc. The highest values of the A-weighted equivalent-continuous SPLs accompanied playing percussion, wind brass instruments (saxophone, trumpet, trombone, tuba, and horn) and wooden wind instruments (bassoon, flute, oboe, and clarinet).

According to the replies presented in the questionnaire, the students devoted to instrument playing on average 27.2 ± 14.4 h weekly, including 16.5 ± 8.2 h of individual playing and 7.6 ± 6.2 h of team playing. In turn, the A-weighted equivalent-continuous SPLs measured during solo playing and team playing remained in the range of 74–107 dB and 80–99 dB, respectively. Subsequently, the personal weekly noise exposure levels (L_EX,w_) determined on that basis for individual students ranged from 75 to 106 dB (M ± SD: 86.8 ± 6.3 dB, median: 84.9 dB).

**Figure 2:**
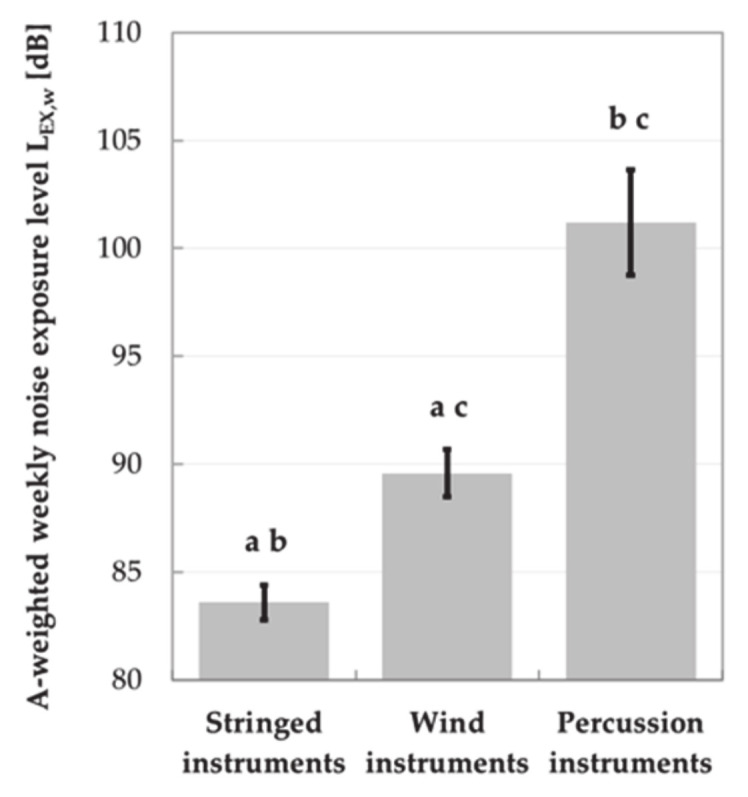
A-weighted weekly noise exposure levels (mean (M) ± 95% confidence intervals (95% CI)) in subgroups of students playing stringed, wind and percussion instruments. Significant differences between pairs of subgroups of music students are marked with a, b or c.

There were significant differences in the L_EX,w_ levels between students playing instruments belonging to different families. The highest values of weekly noise exposure levels occurred in case of students playing percussion instruments, while the lowest those playing the stringed instruments (Figure 2). However, neither gender nor age had a significant impact on student exposure to music.

**Figure 3:**
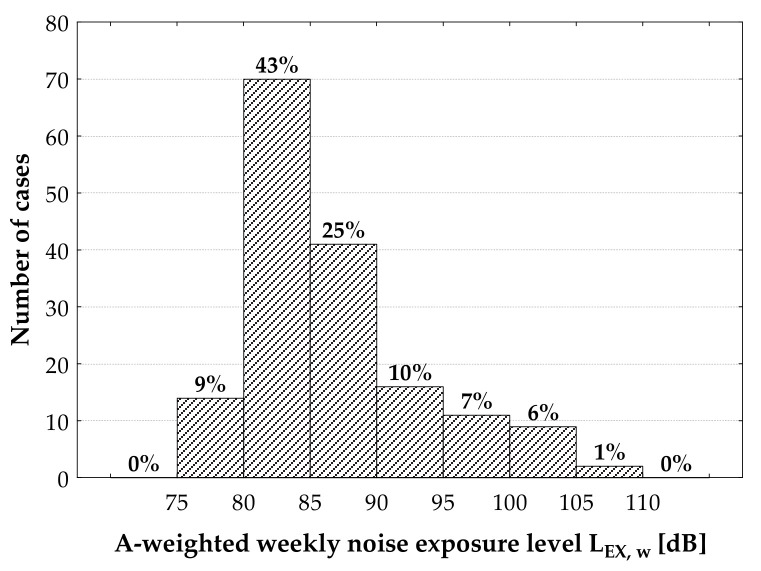
Distribution of the A-weighted weekly noise exposure levels in the music students.

Generally, nearly a half (48.5%) of the study subjects were exposed to music noise at the L_EX, w_ levels exceeding the Polish maximum admissible intensity (MAI) value of noise in the working environment (L_EX, w_ = 85 dB), while 29.4% were exposed to the L_EX, w_ levels above 87 dB, i.e., exposure limit value specified by 2003/10/EC (Figure 3) [33,34]. However, only 9.2% (95% CI: 5.6–14.8%) of responding students declared the use (presently or in the past) of hearing protective devices (mainly ear plugs), while 47.2% (95% CI: 39.7–54.9%) declared to use them in the future.

### 3.3. Results of Hearing Examinations

#### 3.3.1. Pure-Tone Audiometry

The results of audiometric tests carried out in 163 music students (326 ears,) and 67 subjects (134 ears) from the control group are presented in Table 3. All subjects had symmetrical hearing (i.e., mean hearing threshold levels for each ear occurred within 10 dB HL of each other). There were no significant differences in the mean HTLs between their right and left ears for almost all analyzed frequencies, excluding 1 kHz (only in case of music students) and/or 4 kHz. Mean HTL of music students at 1 kHz was higher (worse) for the right ear as compared to the left ear, while a reverse relation was observed in both groups for 4 kHz.

Over half of the music students (62.0%, 95% CI: 54.3–69.1%) and the control group (62.7%, 95% CI: 50.7–77.3%) had bilateral normal hearing (HTLs between 0.25 and 8 kHz ≤ 20 dB HL). Furthermore, in both groups, high-frequency hearing loss and speech-frequency hearing loss were only noted in a few percent of analyzed audiograms (Table 4). On the other hand, typical noise-induced notches at 4 or 6 kHz (of ≥15 dB HL depth) were found in 13.5% (95% CI: 10.2–17.7%) of music students’ ears and 9.0% (95% CI: 5.1–15.2%) of the control group ears. However, neither proportions of high-frequency loss nor proportions of notched audiograms differed significantly in both groups. A greater percentage of audiograms with speech-frequency hearing loss was observed in the control group (4.5%, 95% CI: 1.1–13.0% vs. 0.6%, 95% CI: 0.0–3.8%, *p* < 0.05) (Table 4).

Generally, hearing threshold levels of the music students were lower (better) as compared to the control group in the frequency range from 250 to 3000 Hz (*p* < 0.05), while above 3000 Hz there were no significant differences between these groups (Table 4). Furthermore, such relationships were observed both when analyzing the PTA results for right and left ears together and separately.

**Figure 4:**
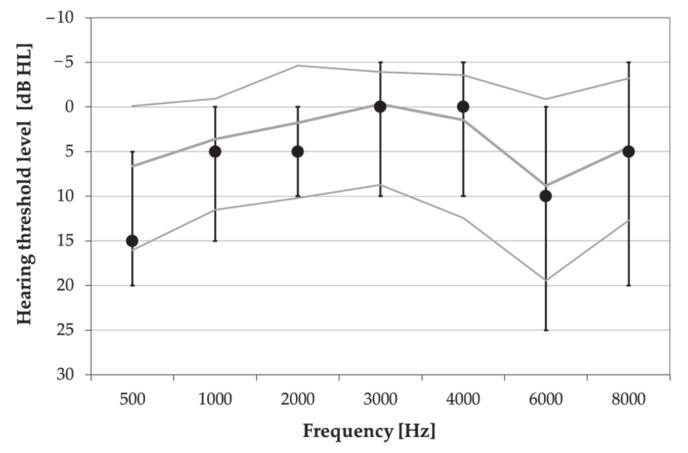
Statistical distribution of hearing threshold levels (HTLs) in the music students compared to HTLs in equivalent—due to age and gender—unscreened reference population (database B4 according to ISO 1999:2013) [14]. Solid lines represent median values of 10th, 50th and 90th percentiles of HTLs in reference population, while dots and whiskers represent 10th, 50th and 90th percentiles of actual HTLs among music students.

A comparison of the music students’ HTLs to statistical distributions of hearing threshold levels compiled from unscreened populations of three typical industrialized societies as specified in ISO 1999:2013 [14] demonstrated that the distribution of their hearing thresholds was most similar to that of the completely unscreened reference population, which also comprises people with occupational exposure to noise, i.e., database B4 (Figure 4).

**Figure 5:**
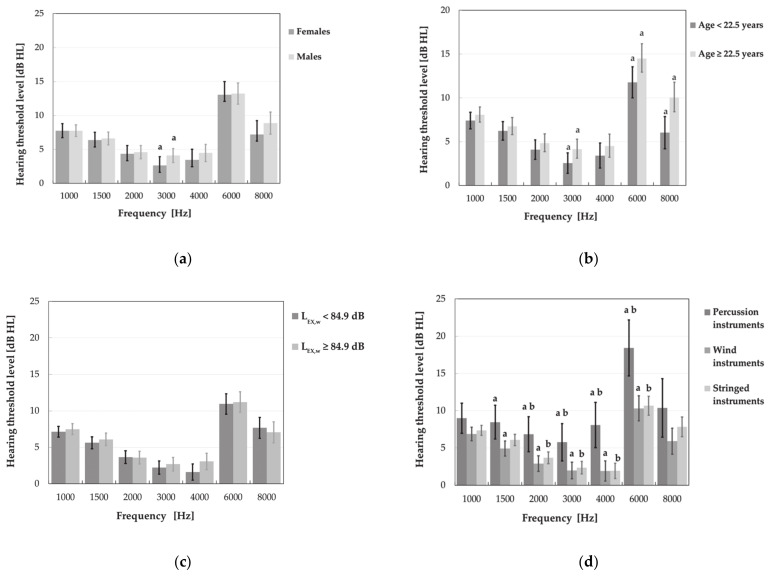
Audiometric hearing threshold levels (M ± 95% CI) determined in various subgroups of music students, i.e., women and men (**a**), younger and older subjects (age < 22.5 and ≥ 22.5 years) (**b**), subjects exposed to lower and higher weekly noise exposure levels (L_EX,w_ < 84.9 and L_EX,w_ ≥ 84.9 dB) (**c**), and subjects playing percussion, wind, and stringed instruments (**d**). Significant differences between subgroups are marked with a or b.

Furthermore, statistical analysis revealed a significant impact of the type of instrument, age and gender on the music students’ hearing threshold levels (Figure 5). Subjects playing the percussion instruments showed higher HTLs as compared to those playing wind or stringed instruments in the frequency range of 1.5–6 and 2–6 kHz, respectively (*p* < 0.05) (Figure 5d). Older subjects (age > 22.5 years) had higher HTLs than younger ones (age ≤ 22.5 years) at 3, 6 and 8 kHz (Figure 5b), while males vs. females—only at 3 kHz (Figure 5c). However, no significant impact of the weekly noise exposure level on the PTA results was noted.

#### 3.3.2. Otoacoustic Emissions

TEOAEs were present bilaterally in all analyzed frequency bands according to the criterion of reproducibility >60% in 75.5% of music students, while considering the signal-to-noise ratio > 6 dB in 33.7% of them. As regards the reproducibility of total response and SNR, the aforesaid criteria were met in 98.8 and 82.8% of the study group, respectively. In turn, the DPOAEs were considered as present in both ears and all analyzed frequencies in 76.1% of them. OAEs were also present in similar (or greater but not significantly) percentages of subjects from the control group (Table 5).

On the other hand, about one-quarter of music students exhibited absent DPOAEs for at least one frequency in at least one ear. The absence of TEOAEs (in one or two ears for at least one frequency band) according to reproducibility criterion was also noted in one-quarter of music students, while based on SNR criterion in 66.1% of them (Table 5).

Generally, insignificantly smaller (or similar) proportions of the control group, exhibited absence of otoacoustic emission as compared to the music students’ group (Table 6). However, significant differences between groups were only found when analyzing the absence of OAEs in individual bands or frequencies.

**Figure 6:**
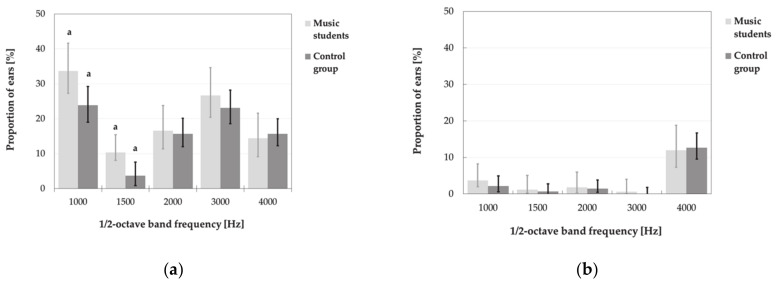
Proportion (with 95% CI) of absent TEOAEs in the music students and in the control group according to the criteria of SNR≤ 6 dB (**a**) and reproducibility ≤ 60% (**b**). Significant differences between groups are marked with a.

**Figure 7:**
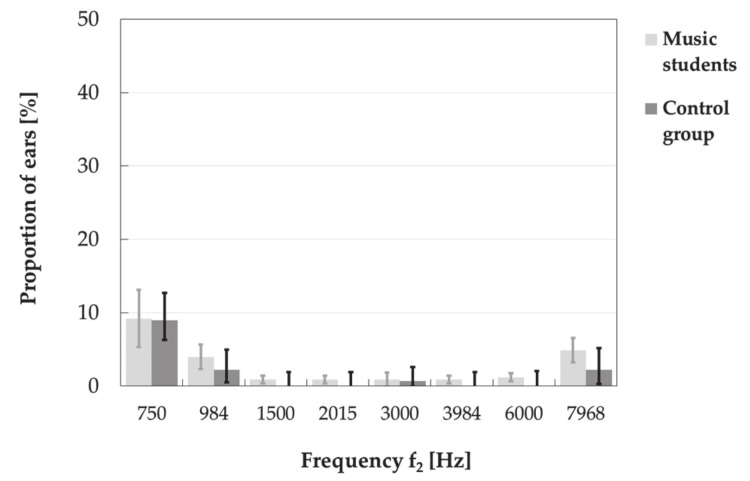
Proportions (with 95% CI) of absent DPOAEs in the music students and in the control group according to the criterion of SNR ≤ 6 dB.

It turned out that the TEOAE responses (in the frequency bands of 1000 and 1500 Hz) were more often absent (due to SNR criterion) in the music students than in the control group (Figure 6). However, basically, the DPOAE responses were mostly absent at frequencies of 750, 984 and 7968 Hz (Figure 7), while the TEAOE signals in ½-octave bands of 1000 and 3000 Hz (due to SNR ≤ 6 dB) or 4000 Hz (considering reproducibility ≤60% (Figure 6).

Results of TEOAE and DPOAE measurements in the group of music students and in the control group are summarized in Table 7 and Table 8, respectively.

Generally, there were no significant differences between music students and control group in case of the majority of TEOAE parameters, excluding signal-to-noise ratio. It turned out that SNR in the frequency band of 1000 Hz was significantly lower (worse) in music students in comparison with the control group, but only if the data concerning both ears were included in analysis. No significant differences were noted between the groups in TEOAE amplitudes after exclusion from analysis responses which did not reach SNR > 6 dB (*p* > 0.05) (Table 7).

In case of the DPOAE testing, the music students proved to be significantly lower (worse), as compared to the control group, mean values of the signal-to-noise ratios at f_2_ frequencies of 984, 6000 and 7969 Hz as well as lower mean values of emissions amplitudes at 6000 and 7969 Hz. The latter differences were also significant, after rejection of cases which did not meet the criterion of SNR > 6 dB.

Furthermore, the aforesaid relations between music students and the control group were visible both when analyzing DPOAEs in left and right ears together and separately. For the other frequencies, no significant differences were observed between the groups (Table 8).

**Figure 8:**
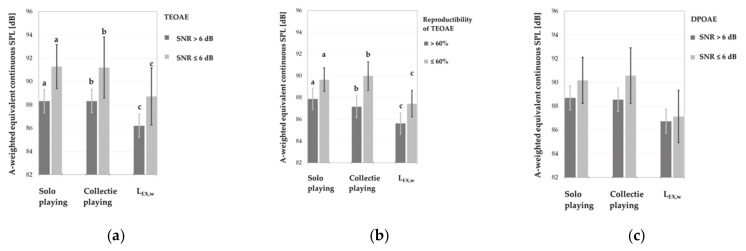
Noise exposure levels (M ± 95% CI) in various subgroups of music students with absent (in at least one ear for at least one band/frequency) and present (bilaterally in all analyzed bands/frequencies) OAEs, i.e., subjects with absent and present TEOAEs with respect to SNR (**a**) and reproducibility values (**b**), and subjects with absent and present DPOAEs according to SNR values (**c**). Significant differences between subgroups of music students with absent TEOAEs (due to SNR ≤ 6 dB or reproducibility ≤ 60%) and present TEOAEs (due to SNR > 6 dB and reproducibility > 60%) are marked with a, b or c.

Analysis of the absence and presence of TEOAEs and DPOAEs among music students showed that the subgroups of students with absent emissions (for at least one frequency/band in at least one ear) were exposed to higher A-weighted equivalent continuous SPLs during both solo and collective playing instruments (Figure 8). However, differences between these noise levels reached statistical significance only for the results of TEOAE testing. Moreover, in the latter case, the resultant values of weekly noise exposure levels were also considerably higher in subjects with absent otoacoustic emissions (Figure 8).

There were no significant differences between the aforesaid subgroups of music students in exposure to recreational noise, e.g., due to frequently attended loud music concerts, spending spare time in pubs, clubs, having noisy hobbies, and frequent listening to personal media players via headphones, etc. In addition, those groups did not differ in age or period of instruments playing. However, men prevailed among the subgroup of students whose TEOAE responses did not reach SNR > 6 dB (60.2%, 95% CI: 50.7–68.9% vs. 26.9%, 95% CI: 17.7–38.6%, *p* < 0.05) or reproducibility > 60% (75.0%, 95% CI: 59.6–85.9% vs. 43.1%, 95% CI: 34.7–51.9%, *p* < 0.05). Meanwhile, males were equally numerous as women in subgroups with absent and present DPOAEs.

**Figure 9:**
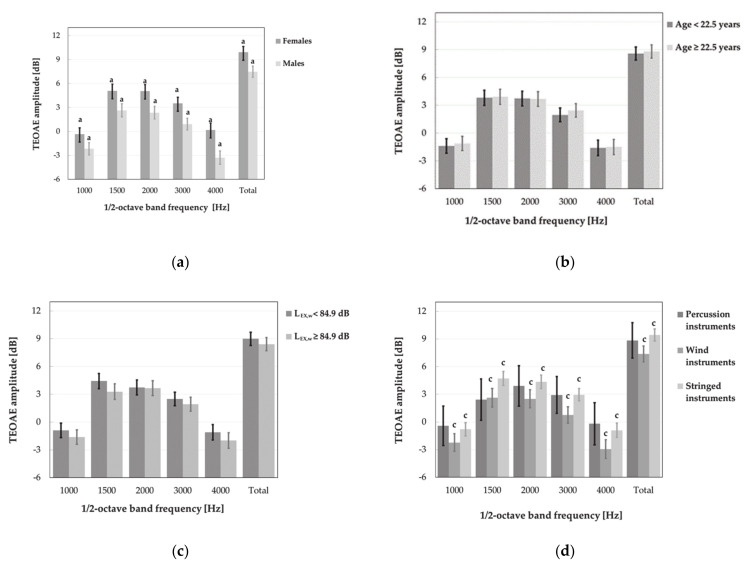
Amplitudes of TEOAEs (M ± 95% CI) registered in various subgroups of music students, i.e., women and men (**a**), younger and older subjects (aged < 22.5 and ≥22.5 years) (**b**), subjects exposed to lower and higher weekly noise exposure levels (L_EX,w_< 84.9 and L_EX,w_ ≥ 84.9 dB) (**c**), and playing percussion, wind and stringed instruments (**d**). Significant differences between subgroups are marked with a or c.

**Figure 10:**
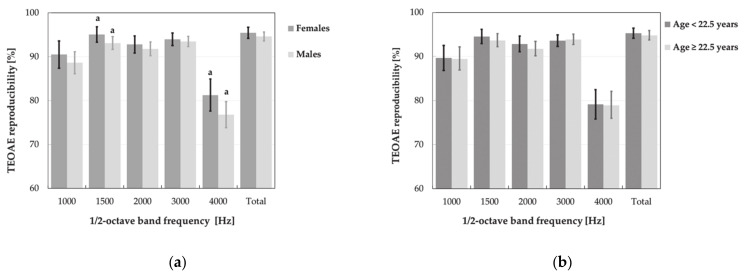

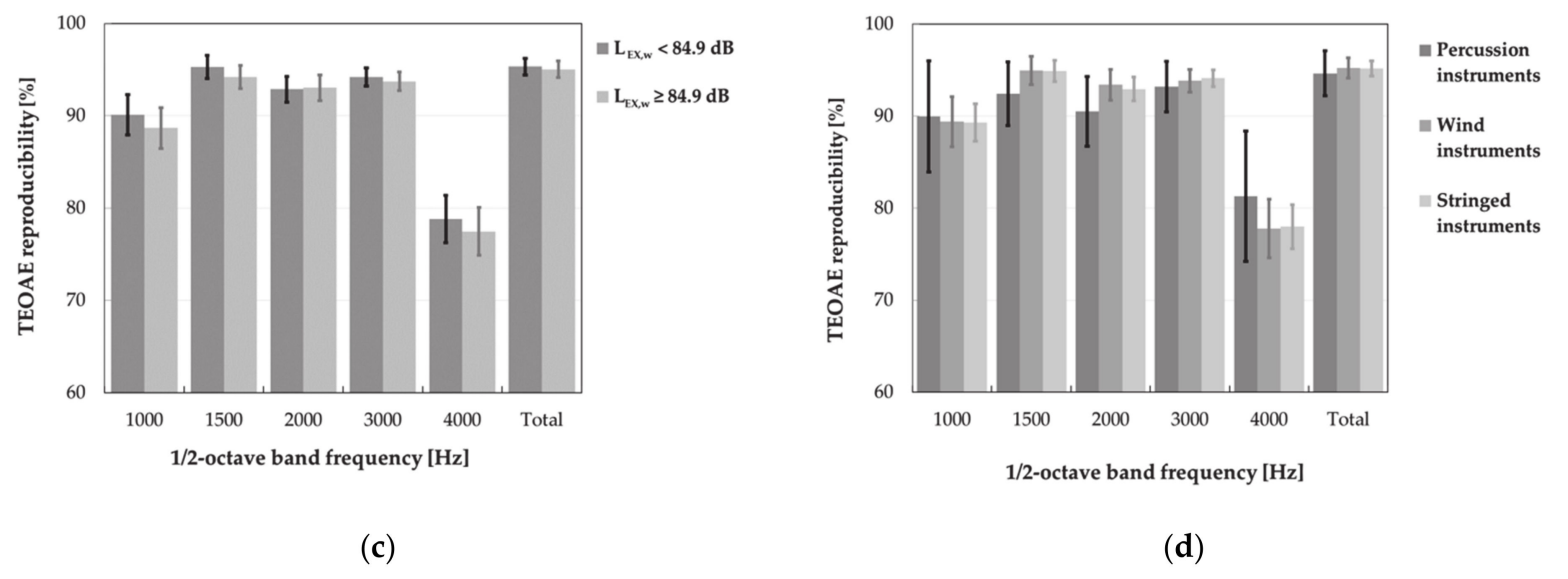
Reproducibility of TEOAEs (M ± 95% CI) measured in various subgroups of music students, i.e., women and men (**a**), younger and older subjects (age < 22.5 and ≥ 22.5 years) (**b**), subjects exposed to lower and higher weekly noise exposure levels (L_EX,w_ < 84.9 and L_EX,w_ ≥ 84.9 dB) (**c**), and playing percussion, wind, and stringed instruments (**d**). Significant differences between subgroups are marked with a.

**Figure 11:**
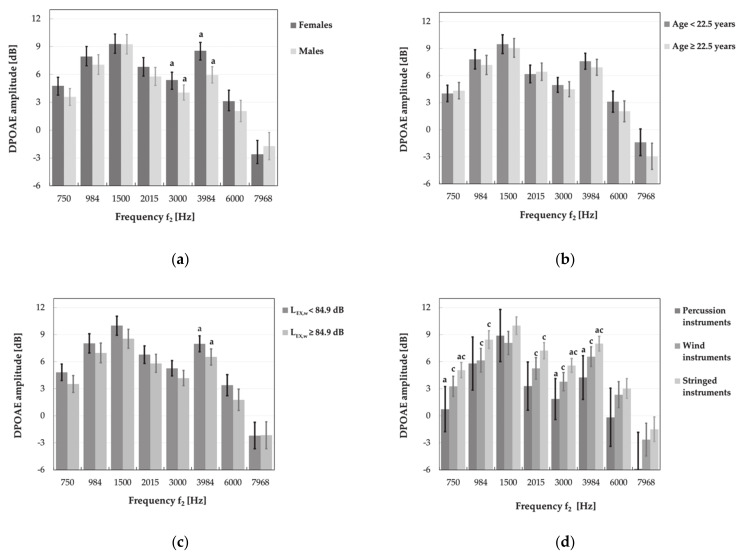
Amplitudes of DPOAEs (M ± 95% CI) measured in various subgroups of music students, i.e., women and men (**a**), younger and older subjects (aged < 22.5 and ≥22.5 years) (**b**), subjects exposed to lower and higher weekly noise exposure levels (L_EX,w_ < 84.9 and ≥ 84.9 dB) (**c**), and playing percussion, wind, and stringed instruments (**d**). Significant differences between subgroups were marked with a or c.

Further statistical analysis, i.e., main effects ANOVA with age, gender and weekly noise exposure level as explanatory (independent) factors, did not show the significant impact of noise levels on the TEOAE parameters, specifically amplitude and reproducibility of responses (Figure 9 and Figure 10). Instead, the significant main effect of weekly noise exposure level on the DPOAE amplitude at single frequency of 3984 Hz was found. Subjects with higher weekly noise exposure levels (L_EX,w_ ≥ 84.9 dB) showed a reduced amplitude compared to those with lower noise levels (L_EX,w_ < 84.9 dB, *p* < 0.05) (Figure 11). The latter relationship was also significant after the responses which did not meet the criterion of SNR > 6 dB had been rejected from the analysis.

Indirectly, the impact of noise exposure level on measured OAEs was visible when the effects of multiple factors such as age, gender and type of instrument were evaluated simultaneously. It turned out that the type of instrument had a significant impact on the DPOAE and TEOAE amplitudes. Music students playing the percussion instruments achieved lower DPOAE amplitudes at 750, 3000 and 3984 Hz as compared to those playing stringed instruments (*p* < 0.05) (Figure 11d). Instead, playing the wind instruments, as compared to stringed instruments, was associated with lower DPOAE amplitudes for 750, 984, 2015, 3000 and 3984 Hz (*p* < 0.05) (Figure 11d). Furthermore, students playing wind instruments also reached lower, as compared to stringed instruments, mean values of TEOAE amplitudes both for total signal and all frequency bands (*p* < 0.05) (Figure 9d).

After rejection of responses which did not meet the criteria of the TEOAE presence, the latter relationships were still significant (excluding the frequency band of 3000 Hz). In turn, after exclusion cases which did not meet the criterion of the DPOAE presence, significant differences between students playing the percussion and stringed instruments were only observed for the DPOAE amplitude at frequency of 750 Hz, while the differences between wind and stringed instruments players were no longer significant at frequencies of 750 and 984 Hz (for comparison see Figure 11d).

No significant main effect of age on the results of OAE measurements was noted. However, men, compared to women, obtained considerably lower (worse) mean values of DPOAE amplitude (for 3000 and 3984 Hz) as well as the TEOAE amplitude (for total response and all frequency band) and reproducibility (for frequency bands of 1500 and 4000 Hz) (Figure 9a, Figure 10a and Figure 11a).

After rejection of responses that do not reach SNR > 6 dB, the impact of gender on the TEOAE amplitude was no longer significant in the frequency bands of 1000 and 1500 Hz, while, in case of DPOAEs, it remained significant for frequencies of 3000 and 3984 Hz (for comparison see Figure 9a and Figure 11a).

## 4. Discussion

The study was aimed to answer two questions. First, if the music students are at higher risk of development of NIHL than their peers who are not occupationally exposed to noise and do not play music instruments? Second, whether or not the students’ hearing status reflects their exposure to music noise resulting from the studies specificity?

In this study, students’ exposure to music noise was evaluated from the results of sound pressure levels measurements during both solo and collective playing using and the declared time of weekly practice obtained from the questionnaire survey.

It is worth noting that these noise measurements were carried out using acoustic dosimeters worn by music students with microphones mounted high on their shoulders. Therefore, sound pressure levels measured during playing percussion instruments might be slightly elevated due to the reflection of sound back from the head and neck. It may also have increased the probability of artifacts and, for this reason, percussion results should be carefully analyzed to eliminate them [16].

Generally, our measurements involved a differentiated repertoire and diverse situations, including mandatory and additional classes, rehearsals and performances, whereas the calculations involved the energy average values of the A-weighted equivalent continuous SPLs determined for particular types of instruments. Subsequently, the individual weekly noise exposure levels (L_EX, w_) determined on that basis for individual students ranged from 75 to 106 dB. The lowest values of the weekly noise exposure level were observed in stringed instruments players, while the highest in those playing percussion instruments. Thus, the upper action limit value (L_EX, w_ = 85 dB) and the exposure limit value (L_EX, w_ = 87 dB) established in Directive 2003/10/EC [34] were exceeded in 49% and 13% of examined music students, respectively.

For comparison, recently, Washnik et al. [21] carried out full-day measurements in the group of 57 classical music students. Their surveys covered two representative week days (from morning to evening) and included both individual practice and ensemble rehearsals for collegiate student musicians.

It appeared that almost half (28/57) of the study subjects exceeded 100% of permissible daily dose according to the National Institute for Occupational Safety and Health (NIOSH) criterion [35]. Furthermore, such situation was repeated twice in 19% students, namely in musicians playing the saxophone, French horn, flute, trombone and trumpet. Fourteen students exceeded 100% dose during large ensemble rehearsals, while eight students—during individual practice sessions. Furthermore, assuming a typical college schedule of classes, the authors concluded that about half of the students exceeded the admissible dose, corresponding to 8-h exposure to noise (sound) of 85 dBA level [21].

In our study, also nearly a half (49%) of the music students were on average exposed for five days a week, 8-h a day to music noise at A-weighted equivalent continuous SPL levels exceeding 85 dB. Thus, despite a different measurement strategy, we obtained similar final outcome of noise exposure evaluation.

In turn, Phillips and Mace [19] measured sound pressure levels (SPLs) among music students during individual classes in specially prepared rooms and found that singers and brass, wind and string players were exposed to averaged sound pressure levels of 87–95 dB—whereas, according to the data collected in this study the A-weighted equivalent continuous sound pressure levels during individual playing remained in the range from 81 to 98 dB (10–90th percentile range).

Generally, results of our noise exposure evaluation due to regular instruments playing are in line with the outcomes of previous investigations, and confirm that young people enrolled in the university music education, likewise professional orchestral musicians, are often exposed to music noise at SPLs exceeding the upper action limit value set up in Directive 2003/10/EC [34], and hence associated with a risk of NIHL [11,12,16].

In order to assess a possible risk of hearing impairment, furthermore, the results of hearing tests among music students were compared with the control group which mainly comprised non-music students and non-musicians not occupationally exposed to noise. However, about one-quarter of them were occasionally exposed to noise during traineeship (internship or apprenticeship).

As mentioned in the introduction, due to the young age of tested subjects and the desire to identify early signs of NIHL, the audiological evaluation was not limited to the standard pure-tone audiometry, but it was extended by measurements of TEOAEs and DPOAEs.

It is worth emphasizing that individual susceptibility to noise, together with the degree of hearing loss, differs greatly among people [36]. It is believed that NIHL is a complex disease resulting from the interaction between environmental and intrinsic factors. Apart from noise, its level, duration and certain characteristics (e.g., impulsiveness and tonality), the former factors are co-exposures to ototoxic substances (organic solvents and heavy metals), co-exposure to noise and vibration; ototoxic drugs (aminoglycosides) and hyperthermia. However, relationships have also been found between some individual factors and NIHL, including smoking, elevated blood pressure, diabetes, cholesterol levels, skin pigmentation, gender and age, and genetic predisposition [36].

The aforesaid individual NIHL risk factors were rare in the music students and the control group. Furthermore, no significant differences between both groups in prevalence of smoking, elevated blood pressure, diabetes, white-finger syndrome, light skin pigmentation, and ototoxic antibiotic treatments were found. Basically, there were also no significant differences between music students and the control group according to age, gender, medical history, physical features and some aspects of lifestyle such as a noisy hobby. Though a greater fraction of music students listened frequently to personal media players, while a greater proportion of those from the control group often used noisy tools in the past.

Majority of the music students (62.4%) and the control group (62.7%) had bilateral normal hearing and both speech frequency hearing loss and high-frequency hearing loss very seldomly occurred in those groups. Nevertheless, audiograms with speech-frequency hearing loss were more often observed in the control group. Furthermore, contrary to our expectations, music students had significantly lower (better) hearing threshold levels in the frequency range from 0.25 to 3 kHz as compared to the control group (*p* < 0.05), while there were no significant differences between these groups in a higher frequency range from 4 to 8 kHz.

Since majority of subjects in this study had hearing threshold levels within normal limits, in order to identify early signs of NIHL, the prevalence of high-frequency notches (at 4 kHz or 6 kHz) in audiograms was also analyzed. It turned out that high-frequency notches were observed more often, but not significantly, in music students as compared to the control group (14.3 vs. 9.0%, *p* > 0.05).

It is worth noting that our previous analysis of audiometric results in music students revealed that the odds ratio of incidence of notched audiograms (OR = 1.070, 95% CI: 1.014–1.130, *p* < 0.05) increased significantly with higher weekly noise exposure levels. Furthermore, these notches occurred more often, but, insignificantly, in musicians playing percussion instruments, as compared to musicians playing stringed or wind instruments [30].

Further statistical analysis showed a significant impact of the type of instrument, age and gender on the music students’ hearing threshold levels. However, no significant impact of the weekly noise exposure level on the PTA results was observed. Older subjects had higher (worse) HTLs than younger ones at 3, 6 and 8 kHz, while males vs. females–only at 3 kHz. Subjects playing the percussion instruments showed higher HTLs as compared to those playing wind or stringed instruments in the frequency range of 1.5–6 and 2–6 kHz, respectively. It is worth emphasizing that the aforesaid hearing threshold elevation observed in percussion players could be a combined effect of high sound pressure levels and short sound attack and the impulsive character of many percussion sounds.

As mentioned earlier, OAEs measurements have been proposed as an objective and sensitive method of detecting preclinical damage of the cochlea due to noise exposure and monitoring early signs of NIHL [22]. Thus, it was hypothesized that music students would have more absent OAEs and worse (reduced) values of their parameters as compared to the control group. Thus, the presence and absence of DPOAEs and TEOAEs as well as the DPOAE and TEOAE parameters were analyzed in study subjects.

In particular, the proportions of subjects exhibited the absence of OAEs for at least one band (or frequency) in at least one ear were evaluated. DPOAEs and TEOAEs were considered as absent if signal-to-noise ratios did not exceed 6 dB. Additionally, in case of TEOAEs, the criterion of reproducibility > 60% was applied.

In this study, about one-quarter of music students exhibited an absence of DPOAEs or TEOAEs due to not meeting the criteria of SNR > 6 dB and reproducibility > 60%, respectively. However, much greater percentage of absent TEOAEs was noted when the criterion of SNR > 6 dB was taken into consideration. It is obvious that the adoption of a less demanding criterion of the OAEs’ presence (e.g., SNR > 0 dB) would result in fewer absent responses.

Basically, there were no significant differences between the music students and subjects from control groups in prevalence of absent OAEs for at least one frequency in at least one ear (see Table 6). Significant differences between groups were only noted when the incidence of absent emissions was considered separately in each band or frequency. It turned out that the TEOAE responses in ½-octave bands of 1000 and 1500 Hz did not meet the criterion of SNR > 6 dB in the greater percentage of music students than the control group (Figure 6a).

Analyzing the TEOAE parameters, a lower mean SNR value was found in the group of music students as compared to the control group, in a frequency band of 1000 Hz (*p* < 0.05), while, in case of other bands and TEOAE parameters, no significant differences between the groups were noted. In turn, in case of the DPOAE testing, significantly lower (worse), as compared to the control group, values of the signal-to-noise ratio (at 6000 and 7969 Hz) and amplitude of responses (at 984, 6000 and 7969 Hz) were observed in the group of music students. Excluding from analyses the TEOAE and DPOAE responses that did not meet the criterion of SNR> 6 dB did not affect the results of the comparisons of signals’ amplitude. Summing up, contrary to standard pure-tone audiometry, both results of TEOAE and DPOAE testing indicated worse hearing in music students in comparison with the controls.

Similar conclusions resulted from some earlier studies. For example, Henning and Bobholz [28] compared the results of DPOAE measurements in the group of 28 college music majors and 35 non-music majors students, aged 18–25 years, with normal hearing within the standard PTA frequency range. There were neither differences in audiometric thresholds between the groups nor in exposure to recreational noise.

As in our studies, the analysis comprised both the presence and absence of DPOAEs and DPOAE amplitudes (for frequencies of 1187, 1500, 1906, 2531, 3031, 3812, 4812 and 6031 Hz frequencies). Although there were no significant differences between both groups in hearing threshold levels and exposure to recreational noise, it appeared that significantly more music majors (7/28) than non-music majors (0/35) exhibited absent DPOAEs (for at least one frequency in at least one ear). Moreover, insignificantly lower by 2–4 dB amplitudes were noted in the right ears of music majors as compared to non-music majors (at frequencies from 3031 Hz to 6031 Hz), as well as in the right ears of music majors playing brass instruments compared to music majors playing nonbrass instruments. In addition, differences, but also insignificant, were noted between women and men who studied at musical faculties, and it appeared that men had worse hearing (lower values of amplitude for frequencies: 3812 Hz, 4812 Hz and 6.031 Hz). Considering a higher incidence of absent DPOAEs among university music majors compared to non-music majors, the authors concluded that that group could exhibit early stages of cochlea impairments [28].

Other earlier research among college music students, based on measurements of otoacoustic emissions, were aimed at evaluation of temporary changes after exposure to music [20,37]

For example, the quoted Gopal et al. [20] attempted to evaluate if students’ participation in 50-min jazz band-based instructional activity was associated with significant temporary worsening of hearing compared to non-music students in regular classroom sessions. For this purpose, noise measurements and hearing tests (i.e., PTA and TEOAE) were carried out twice (i.e., before and just after the classes) in students taking part in jazz band-based practice session (*n* = 14) as well as in non-music students attending a regular classroom session (*n* = 11).

According to results of the latter study, students participating in jazz band-based instructional activity were not only exposed to much higher A-weighted equivalent-continuous SPLs as compared to regular classes (99.5 ± 2.5 vs. 49.9 ± 10.6 dB), but they also exhibited a significant temporary threshold shift (TTS) bilaterally at 4000 Hz as well as a significant decrease in the amplitude of TEOAE after exposure to music—whereas no significant changes were found in the group of students attending regular classes [20].

Otoacoustic emissions were also used by a number of researchers to evaluate noise-induced changes in cochlea among rock musicians. For example, Samelli et al. [38] compared otoacoustic emissions registered in 16 young adult musicians with the control group comprised 16 persons who did not deal professionally with rock music and found statistically significantly lower values of TEOAE amplitude among musicians—no matter if their hearing threshold levels remained within normal limits or not [38].

Maia and Russo [39] analyzing the results of hearing tests in the group of 23 rock musicians aged 21–38 years, with normal hearing, noted the lowest TEOAE amplitudes in 4 kHz band. In turn, Santoni and Fiorini [40], evaluating OAEs among 23 pop/rock male musicians (aged 25–38 years) found that TEOAEs and DPOAEs were not present in 48 and 35% of the subjects, respectively. However, these authors did not specify the criteria of the presence of otoacoustic emissions; therefore, their findings may not be compared with the results of this study.

The results of subsequent studies carried out by Hoydal et al. [41] in the group of 111 rock musicians (aged 16–52 years), comprising PTA and TEOAE testing, pointed to bilaterally worse hearing in the musicians, as compared to the control group, i.e., higher hearing thresholds for 6 kHz and lower value of SNR in 4 kHz band. However, SNR strongly depended on age and hearing threshold within 3–6 kHz frequency. Thus, the authors formulated the conclusion that no significant decrease in SNR in the musicians could be ascertained.

Analysis of presence and absence of OAEs among our study subjects showed that there were no significant differences in age, length of instruments playing and exposure to recreational noise between subjects with present (in all bands) and absent (for at least one frequency band) emissions. It also appeared that music students with absent emissions, in particular TEOAEs, were exposed to music noise at higher levels than those with present emissions. Furthermore, males more frequently than females, exhibited absent TEOAEs, while equally often absent DPOAEs.

Our further analyses of hearing tests’ results in relation to students’ exposure associated with playing instruments showed a significant impact of noise exposure level only in the case of the DPOAE testing. It appeared that students exposed to noise at higher levels (L_EX, w_ ≥ 84.9 dB) exhibited a lower (worse) DPOAE amplitude, but only at frequency of 3984 Hz, as compared to subjects exposed to lower levels (L_EX, w_ < 84.9 dB, *p* < 0.05).

In the next step, a significant influence of the type of instrument, indirectly the impact of the music exposure characterized by the L_EX, w_ level, on measured OAEs was demonstrated. It turned out that lower (worse) values of the TEOAE and DPOAE amplitudes were observed in wind instruments players as compared to students playing stringed instruments. This effect was more distinct in the case of amplitude TEOAEs, because it referred both to the total response and to all analyzed frequency bands, while, in case of DPOAEs, it was noted for a part of analyzed frequencies (i.e., 750, 984, 2015, 3000 and 3984 Hz). Furthermore, students playing percussion instruments exhibited reduced amplitudes of DPOAEs (at frequencies of 750, 3000 and 3984 Hz) in comparison with stringed instruments players.

Earlier, the impact of exposure to excessive sounds on the cochlea in young rock or pop musicians was presented by the above-mentioned Maia and Russo [39], who found statistically significantly lower values of DPOAE amplitudes (for frequencies of 0.75, 1, 4 and 6 kHz) in musicians who played instruments for at least 10 years.

The results of our study presented herein also confirmed the impact of gender, described also in other studies, on the results of hearing tests, i.e., better hearing in women, as compared to men [28,42]. On the other hand, as opposed to some results of research among professional musicians with longer duration of work, they did not confirm the impact of age on registered otoacoustic emissions. However, these results are not surprising because of the tested students’ young age.

## 5. Conclusions

According to the results of our study, nearly a half of music students, due to regular playing instruments, were exposed to music (sounds) at the A-weighted weekly noise exposure level exceeding the upper exposure action value of 85 dB specified in the Directive 2003/10/EC [34]. There were significant differences in noise exposure between students playing instruments belonging to different families. The highest noise levels occurred in the case of students playing percussion instruments, while the lowest in those playing the stringed instruments.

The majority of music students and the control group had bilateral normal hearing in the standard pure-tone frequency range and both speech-frequency hearing and high-frequency hearing loss were very rare in those groups. Music students had significantly lower (better), as compared to the control group, audiometric thresholds in the frequency range of 250–3000 Hz, while there were no significant differences between groups from 4000 to 8000 Hz.

However, DPOAE amplitudes (at 6000 and 7969 Hz) and SNR (at 984, 6000 and 7969 Hz) were significantly reduced in music students, as compared to the control group. Lower values of SNR (in frequency band of 1000 Hz) were also observed among the music students in the case of TEOAE testing. Furthermore, a significantly higher proportion of this group exhibited absent TEOAEs. Thus, contrary to pure-tone audiometry, both TEOAEs and DPOAEs indicated worse hearing among students of music academies as compared to their peers who were not occupationally exposed to noise and did not play musical instruments.

Noise exposure had significant impact on measured DPOAEs. Music students having weekly noise exposure level ≥ 85 dB compared to those with lower than 85 dB exhibited reduced DPOAE amplitude at 3984 Hz.

On the other hand, the type of instrument, and indirectly music exposure characterised by a weak noise exposure level, affected both OAE and PTA results. It turned out that subjects playing the percussion instruments showed higher hearing thresholds as compared to those playing wind or stringed instruments. In turn, both TEOAE and DPOAE results indicated worse hearing in students playing percussion instruments vs. wind instruments, and wind instruments players vs. students playing stringed instruments.

In conclusion, worse results of DPOAE and TEOAE testing in the music students compared to control group suggest the occurrence—in this group—of the early stages of cochlear damage and point to the need to cover them by the hearing protection programme. Further studies are also necessary before firm conclusions can be drawn concerning the risk of hearing loss among music students due to playing instruments.

## Figures and Tables

**Table 1:** Sound pressure levels (SPLs) measured in the music students during individual practice.

Instrument	A-Weighted Equivalent Continuous SPL [dB]	A-Weighted Maximum SPL [dB]	C-Weighted Peak SPL [dB]
	M ± SD [L]/Me	M ± SD/Me	
Percussion (*n* = 18)	98.7 ± 7.8 [107.0]/95.9	106.4 ± 14.7/109.0	133.7 ± 7.2/133.1
Saxophone (*n* = 9)	96.8 ± 1.7 [97.1]/97.1	114.7 ± 10.4/110.3	122.9 ± 5.1/122.5
Flute (*n* = 6)	94.7 ± 4.1 [96.4]/93.9	107.1 ± 2.2/106.9	122.1 ± 3.0/121.6
Trumpet (*n* = 11)	94.2 ± 2.8 [95.0]/93.9	109.0 ± 7.2/107.5	123.7 ± 10.2/123.9
Bassoon (*n* = 5)	93.9 ± 0.9 [93.9]/93.7	100.9 ± 1.4/101.6	114.6 ± 0.7/114.5
Horn (*n* = 5)	93.4 ± 3.2 [94.1]/94.8	107.9 ± 1.2/107.5	122.0 ± 1.5/122.0
Tuba (*n* = 2)	93.0 ± 0.1 [93.0]/93.0	103.7 ± 3.7/103.7	129.3 ± 0.6/129.3
Trombone (*n* = 7)	92.9 ± 5.1 [95.3]/91.8	105.5 ± 4.9/107.5	123.6 ± 4.5/123.0
Clarinet (*n* = 7)	90.9 ± 3.5 [92.4]/89.6	103.7 ± 4.2/105.2	121.2 ± 6.3/123.5
Viola (*n* = 4)	88.1 ± 2.5 [88.5]/88.7	99.8 ± 4.0/100.4	125.7 ± 8.2/121.8
Oboe (*n* = 3)	87.1 ± 0.7 [87.1]/86.9	98.5 ± 0.4/98.3	121.4 ± 10.1/122.9
Accordion (*n* = 6)	86.7 ± 4.4 [88.5]/85.9	108.0 ± 11.8/105.1	125.7 ± 13.3/120.1
Violin (*n* = 20)	85.8 ± 4.2 [87.4]/86.0	97.7 ± 3.7/97.7	116.3 ± 18.1/118.4
Harp (*n* = 4)	85.5 ± 0.6 [85.5]/85.5	99.8 ± 3.0/100.8	123.7 ± 4.7/122.3
Organ (*n* = 1)	83.5 ± 0.0 [83.5]/83.5	94.5 ± 0.0/94.5	106.7 ± 0.0/106.7
Cello (*n* = 6)	83.3 ± 4.7 [85.2]/83.4	97.1 ± 6.9/97.8	123.0 ± 12.1/123.7
Double bass (*n* = 8)	82.7 ± 5.2 [86.1]/82.1	93.6 ± 4.1/92.6	120.2 ± 8.6/121.0
Piano (*n* = 12)	82.0 ± 2.8 [82.7]/82.4	94.4 ± 5.4/93.9	123.8 ± 8.6/123.0
Guitar (*n* = 2)	73.0 ± 4.2 [73.9]/73.0	89.3 ± 1.2/89.3	119.4 ± 4.2/119.4
Total (*n* = 136)	89.9 ± 7.4 [99.1]/90.3	102.5 ± 9.5/102.2	123.0 ± 10.9/123.2

M—arithmetic mean; SD—standard deviation; *n*—number of noise samples; L—energy average of the *n* samples of measured A-weighted equivalent continuous SPL; Me—median.

**Table 2:** Sound pressure levels (SPLs) measured in the music students during group practice.

Instrument	A-Weighted Equivalent Continuous SPL [dB]	A-Weighted Maximum SPL [dB]	C-Weighted Peak SPL [dB]
	M ± SD [L]/Me	M ± SD/Me	
Trumpet (*n* = 13)	98.6 ± 2.0 [99.1]/98.6	115.2 ± 7.1/112.8	130.4 ± 7.8/127.2
Saxophone (*n* = 10)	98.1 ± 3.3 [99.2]/98.3	111.0 ± 7.7/109.3	129.6 ± 8.5/130.8
Trombone (*n* = 11)	96.8 ± 3.2 [98.0]/95.7	104.5 ± 31.9/110.4	132.4 ± 8.6/129.6
Horn (*n* = 3)	94.8 ± 2.0 [95.1]/95.9	115.1 ± 14.1/107.9	127.0 ± 3.0/127.1
Tuba (*n* = 10)	93.7 ± 2.3 [94.2]/93.7	110.3 ± 3.6/110.2	132.7 ± 9.1/130.0
Percussion (*n* = 8)	91.0 ± 4.4 [93.7]/90.0	112.6 ± 4.1/113.0	136.7 ± 3.4/135.0
Clarinet (*n* = 8)	90.2 ± 1.8 [90.5]/90.1	104.2 ± 3.3/103.8	123.8 ± 4.7/122.3
Flute (*n* = 9)	88.8 ± 5.1 [91.3]/89.8	102.6 ± 6.3/102.8	122.2 ± 9.0/122.4
Bassoon (*n* = 3)	87.8 ± 5.7 [90.4]/85.6	98.9 ± 5.5/96.5	118.7 ± 2.2/117.6
Piano (*n* = 13)	86.6 ± 5.2 [90.5]/85.2	100.6 ± 6.2/100.7	127.4 ± 9.2/126.3
Oboe (*n* = 8)	86.3 ± 2.1 [86.7]/85.8	100.8 ± 3.6/100.5	129.1 ± 9.0/130.1
Violin (*n* = 17)	84.6 ± 3.4 [85.8]/84.5	98.4 ± 3.2/99.3	124.8 ± 6.7/126.0
Viola (*n* = 12)	84.0 ± 2.3 [84.6]/83.9	97.9 ± 2.9/98.3	125.0 ± 8.4/127.4
Accordion (*n* = 4)	80.3 ± 5.8 [82.6]/81.0	98.9 ± 3.3/98.1	128.9 ± 4.6/127.9
Cello (*n* = 13)	79.5 ± 2.6 [80.2]/79.2	95.9 ± 3.7/95.2	126.4 ± 6.6/124.8
Double bass (*n* = 7)	78.9 ± 5.4 [82.3]/78.3	98.3 ± 6.6/96.3	127.1 ± 3.2/127.4
Guitar (*n* = 7)	78.8 ± 9.7 [90.3]/73.4	94.0 ± 7.7/93.1	123.2 ± 7.7/124.7
Total (*n* = 157)	88.4 ± 7.6 [93.8]/87.9	103.3 ± 11.5/102.8	127.7 ± 8.1/127.3
Trumpet (*n* = 13)	98.6 ± 2.0 [99.1]/98.6	115.2 ± 7.1/112.8	130.4 ± 7.8/127.2
Saxophone (*n* = 10)	98.1 ± 3.3 [99.2]/98.3	111.0 ± 7.7/109.3	129.6 ± 8.5/130.8
Total (*n* = 136)	89.9 ± 7.4 [99.1]/90.3	102.5 ± 9.5/102.2	123.0 ± 10.9/123.2

Abbreviations as in Table 1.

**Table 3:** Hearing threshold levels (M ± SD) determined in the group of music students and in the control group.

Frequency [Hz]	Music Students	Control Group	Music Students	Control Group	Music Students	Control Group
	Both Ears		Left Ear		Right Ear	
	Hearing Threshold Level [dB HL]					
250	13.6 ± 6.0 ^a^	16.7 ± 6.3 ^a^	13.5 ± 5.8 ^a^	17.1 ± 7.0 ^a^	13.8 ± 6.3 ^a^	16.3 ± 5.5 ^a^
500	13.3 ± 5.5 ^a^	14.7 ± 5.2 ^a^	13.1 ± 5.5 ^a^	15.0 ± 4.9 ^a^	13.4 ± 5.5	14.3 ± 5.4
1000	7.3 ± 4.7 ^a^	8.7 ± 6.0 ^a^	6.8 ± 4.5 ^a b^	8.7 ± 6.2 ^a^	7.9 ± 4.9 ^b^	8.7 ± 5.7
1500	5.9 ± 5.3 ^a^	8.2 ± 6.3 ^a^	6.1 ± 5.6 ^a^	8.3 ± 6.8 ^a^	5.6 ± 5.0 ^a^	8.1 ± 5.8 ^a^
2000	3.6 ± 5.5 ^a^	5.6 ± 6.0 ^a^	3.7 ± 5.8 ^a^	6.1 ± 6.5 ^a^	3.5 ± 5.3 ^a^	5.0 ± 5.4 ^a^
3000	2.5 ± 5.9 ^a^	4.7 ± 7.4 ^a^	2.4 ± 5.9 ^a^	5.1 ± 8.5 ^a^	2.5 ± 6.0 ^a^	4.4 ± 6.1 ^a^
4000	2.3 ± 7.2	2.9 ± 7.1	3.2 ± 7.3 ^b^	4.2 ± 8.4 ^c^	1.5 ± 7.1 ^b^	1.6 ± 5.4 ^c^
6000	11.1 ± 9.0	11.0 ± 9.9	11.3 ± 8.9	12.2 ± 11.3	10.9 ± 9.1	9.9 ± 8.2
8000	7.4 ± 9.4	8.1 ± 9.8	6.9 ± 9.7	8.1 ± 10.4	7.9 ± 9.0	8.1 ± 9.2

^a^ Significant differences between music students and control group (*p* < 0.05). ^b^ Significant differences between the right and left ears of music students (*p* < 0.05). **^c^** Significant differences between the right and left ears of control group (*p* < 0.05).

**Table 4:** Proportions (with 95% confidence intervals (95% CI)) of speech-frequency hearing loss, high-frequency hearing loss and notched audiograms in the music students and in the control group.

Pure Tone Audiometry	Music Students	Control Group
	Proportion (95% CI) [%]	
Speech-frequency hearing loss		
right ear	0.6 (0.0–3.7)	1.5 (0.0–8.8)
left ear	0.6 (0.0–3.7) ^a^	4.5 (1.1–13.0) ^a^
bilateral	0.0 (0.0–2.8)	1.5 (0.0–8.8)
total	0.6 (0.0–2.3) ^a^	3.0 (0.9–7.8) ^a^
High-frequency hearing loss		
right ear	2.5 (0.8–6.4)	1.5 (0.0–8.8)
left ear	1.8 (0.4–5.6)	6.0 (2.0–14.9)
bilateral	0.6 (0.0–2.3)	3.0 (0.9–7.8)
total	2.1 (0.9–4.4)	3.7 (1.4–8.7)
High- frequency notching		
right ear at 4 or 6 kHz	13.5 (9.1–19.7)	6.0 (2.0–14.9)
left ear at 4 or 6 kHz	13.5 (9.1–19.7)	11.9(6.0–22.2)
bilateral at 4 or 6 kHz	4.9 (2.4–9.5)	1.5 (0.0–8.8)
total at 4 or 6 kHz	13.5 (10.2–17.7)	9.0 (5.1–15.2)

^a^ Significant differences between music students and control group (*p* < 0.05).

**Table 5:** Proportion (with 95% CI) of present TEOAEs and DPOAEs in the music students and in the control group.

Criterion of Presence of OAEs	Control Group	Music Students	Control Group	Music Students
	Proportion of Ears (95% CI) [%]		Proportion of Persons ^a^ (95% CI) [%]	
TEOAE – total response SNR > 6 dB reproducibility > 60%	91.0 (84.8–94.9) 100.0 (96.6–100.0)	86.8 (82.7–90.1) 99.4 (97.6–100.0)	88.1 (77.8–94.0) 100.0 (93.3–100.0)	82.4 (75.8–87.5) 98.8 (95.3–99.9)
TEOAE – all bands SNR > 6 dB reproducibility > 60%	56.0 (47.5–64.1) 85.1 (78.0–90.2)	46.6 (41.3–52.0) 85.3 (81.0–88.7)	46.3 (34.9–58.1) 76.1 (64.5–84.8)	33.7 (26.9–41.3) 75.5 (68.3–81.4)
DPOAE – all frequencies SNR > 6 dB	88.1 (81.3–92.6)	84.4 (80.0–87.9)	82.1 (71.0–89.5)	76.1 (68.9–82.0)

OAEs—otoacoustic emissions. TEOAEs—transient-evoked otoacoustic emissions. DPOAEs—distortion-product otoacoustic emissions. SNR—signal to noise ratio. ^a^ People with bilaterally present otoacoustic emissions.

**Table 6:** Proportion (with 95% CI) of absent TEOAEs and DPOAEs in the music students and in the control group.

Criterion of Absence of OAEs	Control Group	Music Students	Control Group	Music Students
	Proportion of Ears (95% CI) [%]		Proportion of Persons ^a^ (95% CI) [%]	
TEOAE–for at least one frequency band				
SNR ≤ 6 dB reproducibility ≤ 60%	44.0 (35.9–52.5) 14.2 (9.2–21.2)	53.4 (48.0–58.7) 14.7 (11.3–19.0)	53.7 (41.9–65.1) 23.9 (15.2–35.5)	66.1 (58.5–72.8) 24.5 (18.6–31.7)
DPOAE–for at least one frequency				
SNR ≤ 6 dB	11.9 (7.4–18.7)	17.3 (13.6–21.8)	17.9 (10.5–29.0)	25.5 (19.4–32.7)

Abbreviations as in Table 5. ^a^ People with absent otoacoustic emissions for at least one ear.

**Table 7:** Results of TEOAEs (M ± SD) measured in the music students and in the control group.

Frequency [Hz]	Both Ears		Left Ear		Right Ear	
	Music Students	Control Group	Music Students	Control Group	Music Students	Control Group
Amplitude [dB]						
1000	−1.3 ± 5.1	−0.3 ± 5.0	−1.7 ± 5.2 ^b^	−0.6 ± 5.1	−0.9 ± 4.9 ^b^	0.0 ± 4.9
1500	3.8 ± 5.4	4.9 ± 5.2	3.5 ± 5.3 ^b^	4.7 ± 5.2 ^c^	4.2 ± 5.6 ^b^	5.0 ± 5.3 ^c^
2000	3.7 ± 5.3	4.1 ± 5.5	3.7 ± 5.2	3.7 ± 5.8	3.7 ± 5.4	4.6 ± 5.2
3000	2.2 ± 4.9	2.2 ± 5.0	2.2 ± 4.6	2.4 ± 5.2	2.2 ± 5.2	2.1 ± 4.9
4000	−1.6 ± 5.6	−1.7 ± 5.0	−1.6 ± 5.6	−1.8 ± 5.0	−1.6 ± 5.7	−1.6 ± 5.0
Total response	8.7 ± 4.7	9.2 ± 4.8	8.5 ± 4.5	9.1 ± 4.9	8.9 ± 4.9	9.4 ± 4.7
Signal-to-noise ratio [dB]						
1000	8.4 ± 4.8 ^a^	9.4 ± 4.8 ^a^	8.1 ± 5.1	9.2 ± 4.9	8.7 ± 4.6	9.6 ± 4.6
1500	12.5 ± 5.2	13.4 ± 4.8	12.2 ± 5.1	13.3 ± 4.8	12.8 ± 5.3	13.4 ± 4.9
2000	11.1 ± 4.9	11.4 ± 5.1	11.1 ± 4.8	10.8 ± 5.3	11.1 ± 5.1	11.9 ± 4.8
3000	9.0 ± 4.7	8.9 ± 4.7	8.9 ± 4.4	8.9 ± 4.8	9.0 ± 5.0	8.8 ± 4.7
4000	9.6 ± 3.9	9.2 ± 3.8	9.5 ± 4.0	9.3 ± 3.9	9.8 ± 3.8	9.1 ± 3.7
Total response	11.4 ± 4.4	11.9 ± 4.4	11.3 ± 4.2	11.7 ± 4.4	11.6 ± 4.5	12.0 ± 4.3
Reproducibility [%]						
1000	89.4 ± 14.0	92.2 ± 10.0	87.9 ± 16.3 ^b^	92.0 ± 10.5	90.8 ± 11.0 ^b^	92.4 ± 9.4
1500	94.7 ± 8.1	95.3 ± 6.8	94.4 ± 9.2	95.5 ± 7.9	95.1 ± 6.8	95.2 ± 5.6
2000	92.9 ± 8.8	93.1 ± 9.5	92.6 ± 9.2	91.9 ± 12.2	93.2 ± 8.3	94.2 ± 5.3
3000	94.0 ± 6.4	93.6 ± 5.3	93.5 ± 7.7	93.7 ± 5.5	94.4 ± 4.6	93.5 ± 5.1
4000	78.1 ± 16.5	76.8 ± 15.4	77.4 ± 16.5	77.3 ± 14.1	78.9 ± 16.5	76.2 ± 16.7
Total response	95.2 ± 5.7	95.3 ± 4.8	94.8 ± 6.7	95.4 ± 5.0	95.6 ± 4.3	95.3 ± 4.7
Corrected amplitude ^d^ [dB]						
1000	1.6 ± 3.5	1.7 ± 3.8	1.5 ± 3.5	1.3 ± 3.9	1.7 ± 3.5	2.2 ± 3.8
1500	5.0 ± 4.4	5.3 ± 4.8	4.8 ± 4.1 ^b^	5.2 ± 4.6	5.3 ± 4.7 ^b^	5.4 ± 4.9
2000	5.4 ± 3.9	5.8 ± 4.2	5.3 ± 3.9	5.7 ± 4.5	5.5 ± 4.0	5.8 ± 4.0
3000	4.4 ± 3.6	4.2 ± 3.8	4.1 ± 3.5	4.4 ± 4.0	4.7 ± 3.7	4.0 ± 3.6
4000	−0.4 ± 5.1	−0.4 ± 4.2	−0.4 ± 5.1	−0.5 ± 4.3	−0.4 ± 5.0	−0.4 ± 4.2
Total response	9.9 ± 3.6	10.1 ± 4.1	9.7 ± 3.5	9.9 ± 4.3	10.1 ± 3.8	10.2 ± 3.9

^a^ Significant differences between music students and control group (*p* < 0.05). ^b^ Significant differences between the right and left ears of music students (*p* < 0.05). ^c^ Significant differences between the right and left ears of control group (*p* < 0.05). ^d^ TEOAE amplitude after rejection of the responses which do not meet the criterion of SNR > 6 dB.

**Table 8:** Results of DPOAEs (M ± SD) measured in the music students and in the control group.

Frequency f_2_ [Hz]	Both Ears		Left Ear		Right Ear	
	Music Students	Control Group	Music Students	Control Group	Music Students	Control Group
Amplitude [dB]						
750	4.2 ± 5.9	4.5 ± 6.0	4.4 ± 5.6	4.9 ± 5.8	3.9 ± 6.2	4.1 ± 6.1
984	7.5 ± 6.9	8.5 ± 7.1	7.5 ± 6.9	8.9 ± 7.3	7.5 ± 6.9	8.0 ± 6.9
1500	9.3 ± 6.7	10.2 ± 6.1	9.0 ± 7.0	10.1 ± 6.1	9.5 ± 6.4	10.3 ± 6.2
2016	6.3 ± 6.3	7.0 ± 5.5	6.5 ± 6.3	6.9 ± 5.8	6.1 ± 6.3	7.1 ± 5.3
3000	4.7 ± 5.4	4.9 ± 5.0	5.4 ± 4.9 ^b^	4.6 ± 5.3	4.0 ± 5.8 ^b^	5.2 ± 4.7
3984	7.2 ± 5.9	7.6 ± 4.6	7.4 ± 5.2	7.5 ± 4.8	7.0 ± 6.5	7.8 ± 4.4
6000	2.6 ± 7.5 ^a^	4.3 ± 6.1 ^a^	2.3 ± 7.6	3.9 ± 6.5	2.8 ± 7.5	4.7 ± 5.8
7969	−2.2 ± 9.4 ^a^	1.6 ± 8.6 ^a^	−2.8 ± 9.4 ^a b^	1.7 ± 9.1 ^a^	−1.6 ± 9.5 ^a b^	1.5 ± 8.1 ^a^
Signal to noise ratio [dB]						
750	12.2 ± 5.8	12.3 ± 5.5	12.2 ± 5.5	12.6 ± 5.7	12.1 ± 6.1	12.0 ± 5.2
984	17.5 ± 7.2 ^a^	19.2 ± 7.5 ^a^	17.8 ± 7.2 ^a^	19.9 ± 7.7 ^a^	17.1 ± 7.2	18.6 ± 7.3
1500	23.5 ± 7.5	24.9 ± 7.2	23.4 ± 7.6	25.2 ± 7.4	23.6 ± 7.4	24.6 ± 7.1
2016	24.7 ± 7.1	25.4 ± 6.3	24.9 ± 6.9	25.2 ± 6.2	24.5 ± 7.3	25.7 ± 6.4
3000	25.1 ± 6.0	25.2 ± 5.7	25.7 ± 5.6	25.1 ± 6.0	24.6 ± 6.4	25.3 ± 5.5
3984	29.0 ± 6.6	29.7 ± 5.4	29.3 ± 5.9	29.8 ± 5.9	28.6 ± 7.3	29.6 ± 4.7
6000	28.1 ± 7.6 ^a^	30.3 ± 7.0 ^a^	27.7 ± 7.9 ^a^	30.4 ± 7.1 ^a^	28.5 ± 7.3	30.2 ± 6.9
7969	22.6 ± 8.7 ^a^	25.9 ± 7.9 ^a^	22.2 ± 8.6 ^a^	26.0 ± 8.5 ^a^	23.0 ± 8.8 ^a^	25.9 ± 7.4 ^a^
Corrected amplitude ^c^ [dB]						
750	5.3 ± 4.6	5.6 ± 5.0	5.3 ± 4.5	6.0 ± 4.8	5.2 ± 4.8	5.1 ± 5.2
984	8.2 ± 6.0	8.9 ± 6.6	8.2 ± 6.0	9.2 ± 6.9	8.2 ± 6.1	8.6 ± 6.3
1500	9.5 ± 6.2	10.2 ± 6.1	9.4 ± 6.2	10.1 ± 6.1	9.6 ± 6.3	10.3 ± 6.2
2016	6.5 ± 5.8	7.0 ± 5.5	6.8 ± 5.5	6.9 ± 5.8	6.2 ± 6.0	7.1 ± 5.3
3000	4.9 ± 4.9	5.1 ± 4.7	5.6 ± 4.6 ^b^	4.9 ± 4.7	4.3 ± 5.0 ^b^	5.2 ± 4.7
3984	7.5 ± 5.1	7.6 ± 4.6	7.4 ± 5.2	7.5 ± 4.8	7.6 ± 5.0	7.8 ± 4.4
6000	3.0 ± 6.6 ^a^	4.3 ± 6.1 ^a^	2.8 ± 6.5	3.9 ± 6.5	3.2 ± 6.7	4.7 ± 5.8
7969	−1.1 ± 8.3 ^a^	2.2 ± 7.7 ^a^	−1.9 ± 8.5 ^a b^	2.5 ± 7.8 ^a^	−0.3 ± 8.1 ^b^	1.8 ± 7.6

^a^ Significant differences between music students and the control group (*p* < 0.05). ^b^ Significant differences between the right and left ears of music students (*p* < 0.05). ^c^ DPOAE amplitude after rejection of the responses which do not meet the criterion of SNR > 6 dB.

## Data Availability

The data presented in this study are available on request from the corresponding author.
